# Caregiver Perception of the Oral-Health-Related Quality of Life of Children with Special Needs: An Exploratory Study

**DOI:** 10.3390/epidemiologia5030038

**Published:** 2024-08-28

**Authors:** Lidia Gavic, Megi Brekalo, Antonija Tadin

**Affiliations:** 1 Study of Dental Medicine, School of Medicine, University of Split, 21000 Split, Croatia; megi.brekalo@gmail.com (M.B.); atadin@mefst.hr (A.T.); 2 Department of Maxillofacial Surgery, Clinical Hospital Centre Split, 21000 Split, Croatia

**Keywords:** dental caries, oral-health-related quality of life, special needs

## Abstract

Background: Compared to the general population, individuals with special needs tend to have worse oral health, potentially diminishing their quality of life. This study aimed to evaluate the perception of parents and caregivers regarding the effect of oral health on the quality of life of individuals with special needs who received dental treatment under general anaesthesia, as well as the impact on their and their families’ quality of life. Materials and methods: This cross-sectional study involved participants with special needs who had undergone a dental treatment under general anaesthesia. Before the intervention, an oral examination was conducted to count the number of teeth affected by caries. Parents or caregivers filled out a specially designed questionnaire that included sociodemographic information, details about the children’s oral hygiene and dietary habits, and questionnaires on the impact of their child’s oral health on their quality of life (P-CPQ) and the influence of the oral health of children with psychophysical difficulties on the family (FIS). The data collected were analysed both descriptively and using the chi-square test, Fisher’s exact test, Kruskal–Wallis test, and Spearman’s correlation analysis. The level of significance was set at *p* ≤ 0.05. Results: This study involved 42 children (24 females and 18 males) with an average age of 21.14 ± 8.34 years. The average number of carious teeth per participant was 9.74 ± 5.63. About 66% of respondents reported that their children performed oral hygiene with their help, while 9.5% of them did not do so at all. Individuals with a higher number of caries had statistically significantly higher scores on the emotional well-being (*p* = 0.004) and social well-being (*p* = 0.033) subscales of P-CPQ, as well as on the parental emotions subscale of FIS (*p* = 0.020). Also, there was a difference in the number of carious teeth in participants due to unhealthy habits (drinking sweetened beverages, *p* = 0.030) and due to comforting with food (*p* = 0.004). Conclusion: The increase in the number of carious teeth in individuals with special needs has been associated with the quality of life of their families. To address this, it is crucial to promote the prevention of oral health issues by educating individuals with special needs and their caregivers on proper oral hygiene techniques and diets tailored to their specific requirements.

## 1. Introduction

Quality of life (QoL) is a complex and multifaceted concept that cannot be defined by a universally accepted definition [1]. Common factors influencing QoL include personal health (physical, mental, and spiritual), social relationships, educational status, social status, work environment, wealth, sense of security and freedom, autonomy in decision-making, social belonging, and their environment [2].

Understanding the principles of QoL contributes to improved symptom management, patient care, and rehabilitation, making it a significant aspect and goal of research and practice in the fields of medicine and healthcare [3]. Despite the relatively recent emergence of the concept, oral health-related quality of life (OHRQoL) has become an important contribution to clinical practice and dental research. Subjective assessment pertains to individuals’ enjoyment during eating, sleeping, and social interactions, as well as their self-esteem and satisfaction regarding their oral health [4]. Measures of OHRQoL provide crucial insights for assessing the treatment needs of individuals and populations, as well as for making clinical decisions and evaluating interventions, services, and public health programs. Four interconnected domains are used to measure OHRQoL: oral symptoms, functional limitations, social well-being, and emotional well-being [5]. According to the World Health Organization, oral health refers to the condition of the mouth, teeth, and orofacial structures that enables individuals to perform functions such as chewing, breathing, and speaking. It also encompasses psychosocial dimensions such as self-confidence, well-being, and the ability to socialize and work without pain, discomfort, or embarrassment [6]. Scientific research consistently confirms that health begins in the mouth [7,8]. Today, good oral health is not only focused on dental health, but serves as the foundation for overall health and well-being of the entire body. Oral health has potential multi-organ systemic repercussions, ranging from insulin resistance to far more complex complications of the cardiovascular or even nervous system. Consequently, improving oral health could have a significant impact on the body, on the prevention of pathology, and thereby on society as a whole and quality of life [9].

Children and individuals with psychophysical difficulties are those with chronic physical, developmental, behavioural, or emotional conditions that require more complex approaches and healthcare services than individuals without difficulties [8]. These individuals are more likely to experience problems with oral health and have different treatment modalities (more extractions, fewer preventive measures), and they may encounter greater challenges in accessing healthcare services, which can negatively impact their well-being and quality of life [10].

Maintaining oral hygiene requires time, ability, and motivation. Such tasks are more challenging for individuals with special needs due to a lack of the manual dexterity or cognitive skills needed to understand the need for effective oral hygiene. Additionally, other conditions, such as sensory impairment or chronic medical conditions, may further complicate the process [11].

Inadequate education of caregivers about oral hygiene and lack of cooperation from children and adults with special needs lead to chronically poor oral health. Despite not being considered a priority, oral hygiene can impact individuals’ quality of life as it can cause pain and discomfort, sleep disturbances, and/or lack of confidence [12].

The aim of this study was to assess parents’ and caregivers’ perceptions of the impact of their child’s oral health on their quality of life using the Parental-Caregiver Perceptions Questionnaire (P-CPQ) and to evaluate the influence of oral health of children and adults with psychophysical difficulties on the family using the Family Impact Scale (FIS). Additionally, the study aimed to examine the impact of sociodemographic factors, oral status, dietary habits, and oral hygiene practices on the perceptions of parents and caregivers (P-CPQ) and the family (FIS). Additionally, the objectives of the study were to evaluate the level of knowledge of parents/caregivers about oral health and to assess the overall oral health of participants with psychophysical disabilities using the DMFT (Decayed, Missing, Filled Teeth) index.

## 2. Materials and Methods

This cross-sectional study was conducted at the Clinical Hospital Centre Split in the Department of Maxillofacial Surgery from June 2022 to July 2023. The study was approved by the Ethics Committee of the Clinical Hospital Centre Split (Class: 500-03/23-01/146, Reg. No.:2181-147/01/06/LJ.Z.-23-02), and it was conducted in full accordance with the World Medical Association Declaration of Helsinki [13]. During the specified period of the survey’s conduct, 48 patients underwent treatment of oral rehabilitation under general anaesthesia at the Department of Maxillofacial Surgery. All of them were invited to participate in the study, and the purpose and objectives of the research were explained to them. Out of the total, 42 agreed to participate, resulting in a response rate of 87.5%.

The participants were individuals with psychophysical disabilities aged 6 to 40 years who underwent oral cavity rehabilitation under general anaesthesia, as well as their parents/caregivers. A questionnaire was distributed to the parents/caregivers, along with informed consent for participation in the study.

Before the dental treatment under general anaesthesia, a clinical oral examination of the participants was performed by two examiners: a sixth-year dental medicine student and a dentist (specialist in endodontics and restorative dental medicine/paediatric dental medicine specialist).

The following data were recorded in the World Health Organization Oral Health Assessment Form for Children, 2013: the number of teeth with active caries, the number of teeth with fillings, and the number of extracted teeth [14]. The DMFT index was calculated for each participant depending on whether the patient had mixed or permanent dentition. Also, the Significant Caries Index (SiC) was calculated [15]. The SiC index was calculated from one-third of the population with the highest caries results as the mean DMFT for that subgroup, and attention was directed to individuals with the highest caries results in each population. Meanwhile, the parents/caregivers completed a questionnaire divided into three parts. The first part gathered personal and demographic information. The second part of the questionnaire included questions on the oral hygiene and dietary habits of the participants. Additionally, it assessed the presence of parafunctions and previous traumatic dental injuries. Three questions were included in the test about parents’/caregivers’ knowledge on oral hygiene and health. For the third part, the Parental-Caregiver Perceptions Questionnaire (P-CPQ) was used. It consisted of questions related to oral symptoms, functional limitations, emotional well-being, and social well-being [16]. Additionally, the impact of the child’s issues on the family was assessed using the Family Impact Scale (FIS-8) [17]. The FIS-8 included questions related to Parental Emotions, Parental/Family Activity, and Family Conflict. The P-CPQ was divided into four subscales: oral symptoms, functional limitations, emotional well-being, and social well-being. Similarly, the FIS-8 was divided into three subscales: Parental Emotions, Parental/Family Activity, and Family Conflict. All questions referred to the past three months [16,17]. The questionnaires were translated into Croatian by two doctors of dental medicine, and two native English speakers back-translated this version into English. Subsequently, the two versions were synthesized and the final questionnaire was compared to the original English-language instrument. The translation process was overseen by a committee that included two forward translators, two back-translators, and a methodologist. The Croatian translations of the questionnaire demonstrated very good reliability, as evidenced by Cronbach’s alpha coefficients of 0.729 and 0.722, respectively [18].

## 3. Statistical Analysis

All properly completed questionnaires were entered into Microsoft Excel 2021 (Microsoft Corporation, Redmond, Washington, DC, USA), and statistical analysis of the collected data was performed using the SPSS 26 software package (IBM Corp., Armonk, New York, NY, USA). Descriptive statistics were used to process the general and demographic data of the participants, oral cavity status, and the quality-of-life data for children and adults with special needs related to oral health. The normality of data distribution was tested using the Kolmogorov–Smirnov test. Differences in the total scores obtained from the P-CPQ questionnaire were analysed using the chi-square test, Fisher’s exact test, the Kruskal–Wallis test, and Spearman’s correlation analysis. The level of significance was set at *p* ≤ 0.05.

## 4. Results

This cross-sectional study included 42 individuals with special needs (24 females and 18 males). Their ages ranged from 6 to 40 years, with a mean age of 21.14 ± 8.34 years, and they had undergone dental rehabilitation under general anaesthesia. The average number of carious teeth per participant was 9.74 ± 5.63, while the SiC in this study was 17.77. Regarding the accompanying caregivers, the questionnaire was mostly filled out by female individuals, who numbered 38 (90.47%). The demographic data of the participants and caregivers are presented in Table 1. Furthermore, in Table 1, the oral statuses of the participants are shown.

In Table 2, the dietary habits of participants who underwent dental restoration under general anaesthesia are presented. Moreover, the table presents results concerning the number of carious lesions in participants, categorized by their dietary habits.

Table 3 and Table 4 show the oral hygiene habits of the participants and the number of carious lesions with respect to their oral hygiene habits.

In Table 5, the results of the P-CPQ and FIS-8 questionnaires are presented as subscales. The P-CPQ test scores ranged from 7 to 39, with a median value of 21, where a higher score indicates poorer oral health-related quality of life. The FIS-8 test scores ranged from 0 to 22, with a median of 9, where a higher score reflects a greater impact of oral health on functioning.

According to the Kruskal–Wallis test and pairwise analysis, individuals with a higher number of caries also had statistically significantly higher scores on the emotional well-being (*p* = 0.004) and social well-being (*p* = 0.033) subscales. Furthermore, parents of participants with a higher number of caries had higher scores on the parental emotions (*p* = 0.020) subscale of the FIS-8 questionnaire. Spearman’s correlation analysis revealed a positive, statistically significant correlation between the total values of participants’ emotional well-being and unhealthy eating habits in children and adults with psychophysical difficulties (R = 0.325, *p* = 0.047). Oral symptoms positively correlated with functional limitations (R = 0.546, *p* ≤ 0.001) and social well-being (R = 0.347, *p* = 0.033). However, unhealthy eating habits positively correlated with oral hygiene habits (R = 0.048, *p* = 0.011).

## 5. Discussion

The aim of this study was to evaluate the association between the oral health of participants with psycho-physiological difficulties and their oral health-related quality of life. These participants underwent dental rehabilitation under general anaesthesia, and the assessment was carried out using specially designed questionnaires and oral examinations. The condition of hard dental tissues (using the DMFT index), oral hygiene, and dietary habits were determined for all participants through a questionnaire, and the oral health-related quality of life was examined. The study included 42 children and adults with psychophysical difficulties who had undergone dental treatment under general anaesthesia, as well as their caregivers. The most common diagnoses among the participants were autism (26.2%), and moderate and mild intellectual disability (28.6%).

Children with autism spectrum disorder often exhibit serious behavioural struggles, including aggression and self-injury, which can lead to traumatic injuries. Since these patients often have anterior open bite and class II malocclusion, they are more susceptible to dental trauma, especially to the upper central and lateral incisors [19]. In our research, lip, cheek, or tongue biting occurred in 31% of participants with varying frequencies (rarely, sometimes, and often). A study by Yashoda and Puranik investigated lip biting, which was present in 22.9% of autistic children [20].

Bruxism or teeth grinding is a serious psychophysiological disorder and a common clinical issue in dentistry [21]. In this study, teeth grinding was observed in 41.5% of participants. This prevalence is, thus, much higher than that of the healthy population. Namely, in the study conducted by Wetselaar et al. in Dutch adolescents, a prevalence of 4.1% was found for awake bruxism and 7.6% for sleep bruxism [22].

To maintain oral health, it is essential to start with proper oral hygiene habits from birth. Most participants (66.7%) in this study maintained their oral hygiene with the help of their caregivers. Furthermore, 23.8% of participants maintained oral hygiene independently, which collides with the results of the study by Pini et al., where as many as 85% of participants maintained oral hygiene independently [23].

Fluorides are crucial in caries prevention, with studies indicating their significantly greater efficacy when applied locally compared to systemic use. Long-term exposure of teeth to fluoride ions has proven to be the most effective method [24]. In our study, only 4.8% of participants used fluoride toothpaste every day, while 21.4% used it often. Chemical plaque control, including the use of antimicrobial agents like chlorhexidine and xylitol, is crucial for caries prevention [24,25,26]. However, our results indicate that the majority of participants did not use xylitol, and 69% never used additional oral health maintenance aids, which may have had an impact on overall oral health (Table 4).

Given all the problems and limitations faced by individuals with psychophysical difficulties, it is necessary to dedicate additional attention to good oral hygiene. Also, regular dental check-ups are crucial.

In our study, the mean DMFT index was 11.24 ± 6.00, which is consistent with a study from Brazil conducted on 47 individuals with special needs aged 12 to 60, where the DMFT mean was also 11 [23]. The SiC in participants with developmental difficulties in this study was 17.77, which is significantly higher than the results obtained in a study conducted in 2016 in Croatia on patients with autism, where the SiC index at that time was 13 [27].

Oral symptoms comprised the first part of the questionnaire on oral health-related quality of life. Toothache, bad breath, and food impaction between teeth were the most common problems reported by caregivers as occasional issues their children face. Among functional limitations, most children have difficulty biting or chewing hard food. Correlation analysis revealed that oral symptoms were positively correlated with functional limitations (R = 0.546, *p* ≤ 0.001). More than 50% of participants were sometimes irritable in the last three months, as well as fearful/anxious. Consequently, their emotional well-being was affected, which positively correlated with unhealthy dietary habits in participants (R = 0.325, *p* = 0.047). These results may imply that parents/caregivers try to resolve bad moods or crying with sweets or sugary drinks, which can consequently lead to the development of cavities. This further leads to a deterioration of emotional well-being, continuing the vicious cycle.

A child’s medical condition has an impact on their overall well-being, but also affects their environment. Caregivers sometimes face a lack of time for themselves and other family members (28.95%). Also, they are sometimes sleep-deprived (42.11%) due to the child’s condition, requiring extra attention. More than 60% of caregivers believe that their child has never been the cause of arguments/disagreements or financial difficulties in the family.

A statistically significant difference was observed on the emotional well-being (*p* = 0.004) and social well-being (*p* = 0.033) subscales of the P-CPQ questionnaire concerning the DMFT index of the participants.

People with special needs often have difficulty expressing pain, which frequently goes unrecognized because some of the children and individuals with special needs cannot verbally express their pain and problems. However, what parents and caregivers notice are emotional changes [28,29]. From our results, it was observed that children and individuals with a higher number of cavities experienced more frequent negative emotions and were more irritable, which can be attributed to poorer oral health.

Considering the DMFT index of the participants, no differences were observed on the FIS-8 subscale related to family conflicts or the impact on family activities. However, a statistically significant difference was observed on the parental emotions subscale. Caregivers whose wards had more cavities experienced negative emotions (guilt and distress). These results are in accordance with those of the study by Akhter et al., which was conducted on the parents of children with cerebral palsy [30].

This study did not find a correlation between the DMFT index and oral health-related quality of life. Since the study examined caregivers’ perceptions of their children’s quality of life, we cannot conclusively say whether it was affected or not. In contrast, a study from India found a significant correlation between DMFT and oral symptoms and functional limitations in children with autism [20].

The main limitation of this study is the fact that it included only participants for whom, due to physical or psychological limitations, dental treatment could not be performed in an ambulatory setting and general anaesthesia was required. Furthermore, this study was conducted at only one clinical hospital centre, so the results cannot be generalized. In the future, more extensive research should be conducted involving a larger number of patients and multiple centres. Additionally, it would be necessary to include those individuals and children with special needs who are cooperative and can be treated in an outpatient setting. Additionally, one of the limitations of the study is the age range of the participants. Specifically, this study includes patients of various ages, and younger patients may face different adjustment challenges compared to older individuals. Moreover, the education levels of parents can influence their understanding of their child’s needs and the necessary interventions. Higher education levels may lead to a better comprehension of the issues and solutions, resulting in more effective support for the patient. Furthermore, the participants in the study have varying degrees of autonomy, which may impact the study’s results. Therefore, future research should aim to examine this aspect more thoroughly by also considering the degree of patient autonomy.

According to the American Academy of Paediatric Dentistry (AAPD), caregivers are the key factor in maintaining the oral health of people with special needs [31]. Unfortunately, in this study, four participants did not maintain oral hygiene at all, which is concerning. Mechanical plaque removal with a toothbrush and fluoride toothpaste is a fundamental step towards oral health, so the fact that some patients currently do not maintain oral hygiene is particularly concerning.

The results obtained suggest the need for additional education of caregivers on maintaining oral hygiene and using additional means to preserve oral health, as well as the importance of healthy dietary habits in individuals with developmental difficulties. The role of caregivers regarding the integration of oral health care into the daily lives of children and adults is crucial. Through training and education, as well as regular preventive care, oral hygiene is improved, consequently enhancing the quality of life of individuals with special needs.

## Figures and Tables

**Table 1:** The demographic data of the participants and parents/caregivers.

Characteristics		Total (*n* = 42)	Number of Carious Lesions			*p*
			<5 (*n* = 6)	5–10 (*n* = 25)	>10 (*n* = 11)	
Sex	Male	24 (57.1%)	4 (9.5%)	15 (35.7%)	5 (11.9%)	0.650
	Female	18 (42.9%)	2 (4.7%)	10 (23.8%)	6 (14.2%)	
Age (years)		21.10 ± 8.38	19.67 ± 7.44	20.20 ± 7.73	23.91 ± 10.27	0.687
Carious lesions before the treatment		9.71 ± 5.64	3.50 ± 1.22	7.96 ± 1.88	17.09 ± 5.54	**≤0.001**
Fillings before the treatment		5.62 ± 2.73	2.17 ± 0.98	5.60 ± 2.27	7.55 ± 2.54	**≤0.001**
Extractions before the treatment		4.10 ± 9.94	1.33 ± 1.63	2.36 ± 2.15	9.55 ± 7.91	**0.008**
Psychophysical difficulties	Autistic Spectrum Disorders	11 (26.2%)	0 (0%)	10 (23.8%)	1 (2.38%)	**0.017**
	Cerebral Palsy	6 (14.3%)	2 (4.76%)	4 (9.5%)	0 (0%)	
	Down Syndrome	4 (9.5%)	0 (0%)	2 (4.7%)	2 (4.76%)	
	Moderate/Mild Intellectual Disability	12 (28.6%)	0 (0%)	4 (9.5%)	4 (9.52%)	
	Other	9 (21.4%)	4 (9.5%)	5 (11.9%)	4 (9.5%)	
Education of individuals with difficulties	Regular school	6 (14.3%)	4 (9.5%)	2 (4.7%)	0 (0%)	**0.005**
	Specialized institution	21 (50.0%)	1 (2.3%)	13 (30.9%)	7 (16.67%)	
	Home care	15 (35.7%)	1 (2.3%)	10 (23.8%)	4 (9.5%)	
Prior dental treatment under general anaesthesia	Yes	21 (50.0%)	4 (9.5%)	12 (28.5%)	5 (11.9%)	0.671
	No	21 (50.0%)	2 (4.7%)	13 (30.9%)	6 (14.2%)	
Parental/Caregiver’s educational level	Elementary	3 (7.1%)	0 (0%)	1 (2.3%)	2 (4.7%)	**0.017**
	High	27 (64.3%)	3 (7.1%)	18 (42.8%)	6 (14.2%)	
	Higher	10 (23.8%)	1 (2.3%)	6 (14.2%)	3 (7.1%)	
	MSc/PhD	2 (4.7%)	2 (4.7%)	0 (0%)	0 (0%)	
Age of parents/caregivers (years)		53.05 ± 9.94	50.83 ± 6.14	54.48 ± 9.86	51.00 ± 11.84	0.258

The values are presented as whole numbers and percentages. Chi-squared test, Fisher’s exact test, or Kruskal–Wallis test. Significant values are in bold.

**Table 2:** Dietary habits of the participants.

		Number of Carious Lesions			
	Total (*n* = 42)	<5 (*n* = 6)	5–10 (*n* = 25)	>10 (*n* = 11)	*p*
**How often does he/she eat sweets/snacks?**					
Never	4 (9.5%)	0 (0%)	4 (9.5%)	0 (0%)	0.116
Rarely	10 (23.8%)	2 (4.7%)	4 (9.5%)	4 (9.5%)	
Sometimes	14 (33.3%)	3 (7.1%)	9 (21.4%)	2 (4.7%)	
Often	12 (28.57%)	1 (2.3%)	8 (19.0%)	3 (7.1%)	
Very often/Daily	2 (4.8%)	0 (0%)	0 (0%)	2 (4.7%)	
**How often does he/she drink sugary drinks?**					
Never	6 (24.3%)	0 (0%)	6 (14.2%)	0 (0%)	**0.030**
Rarely	10 (23.8%)	0 (0%)	6 (14.2%)	4 (9.5%)	
Sometimes	17 (40.5%)	5 (11.9%)	10 (23.8%)	2 (4.7%)	
Often	7 (16.7%)	1 (2.3%)	3 (7.1%)	3 (7.1%)	
Very often/Daily	2 (4.8%)	0 (0%)	0 (0%)	4 (9.5%)	
**How often do you give your child food to make them feel better when they are upset, angry, or hurt?**					
Never	14 (33.3%)	3 (7.1%)	7 (16.6%)	3 (7.1%)	**0.004**
Rarely	14 (33.3%)	2 (4.7%)	12 (28.5%)	0 (0%)	
Sometimes	7 (16.7%)	0 (0%)	6 (14.2%)	1 (2.3%)	
Often	5 (11.9%)	1 (2.3%)	0 (0%)	2 (4.7%)	
Very often/Daily	2 (4.7%)	0 (0%)	0 (0%)	0 (0%)	
**How often do you reward good behaviour with sweets?**					
Never	15 (35.7%)	2 (4.7%)	11 (26.1%)	2 (4.7%)	0.097
Rarely	8 (19.0%)	0 (0%)	3 (7.1%)	5 (11.0%)	
Sometimes	14 (33.3%)	3 (7.1%)	9 (21.4%)	2 (4.7%)	
Often	4 (9.5%)	1 (2.3%)	2 (4.7%)	0 (0%)	
Very often/Daily	0 (0%)	0 (0%)	0 (0%)	0 (0%)	

The values are presented as a whole number and a percentage. Chi-squared test or Fisher’s exact test. Significant values are in bold.

**Table 3:** The oral habits of the participants.

	Total (*n* = 42)	Number of Carious Lesions			
		<5 (*n* = 6)	5–10 (*n* = 25)	>10 (*n* = 11)	*p*
**Bites lips, cheeks, or tongue?**					
Never	29 (69%)	2 (4.7%)	21 (50%)	6 (14.28%)	**0.019**
Rarely	6 (14.3%)	3 (7.1%)	1 (2.3%)	2 (4.7%)	
Sometimes	5 (11.9%)	0 (0%)	2 (4.7%)	3 (7.1%)	
Often	2 (4.8%)	1 (2.3%)	1 (2.3%)	0 (0%)	
Very often/Daily	0 (0%)	0 (0%)	0 (0%)	0 (0%)	
**Grinds teeth?**					
Never	25 (59.5%)	4 (9.5%)	15 (35.7%)	6 (14.2%)	0.392
Rarely	5 (11.9%)	0 (0%)	5 (11.9%)	0 (0%)	
Sometimes	8 (19.0%)	1 (2.3%)	3 (7.1%)	4 (9.5%)	
Often	4 (4.8%)	1 (2.3%)	2 (4.7%)	1 (2.3%)	
Very often/Daily	0 (0%)	0 (0%)	0 (0%)	0 (0%)	
**Has the patient experienced tooth trauma?**					
Yes	12 (28.6%)	2 (4.7%)	6 (14.2%)	4 (9.5%)	0.724
No	30 (71.4%)	4 (9.5%)	19 (45.2%)	7 (16.6%)	

The values are presented as whole numbers and percentages. Chi-squared test or Fisher’s exact test. Significant values are in bold.

**Table 4:** The oral hygiene habits of the participants.

	Total (*n* = 42)	Number of Carious Lesions			*p*
		<5 (*n* = 6)	5–10 (*n* = 25)	>10 (*n* = 11)	
**Do they brush their teeth with fluoridated toothpaste?**					
Never	13 (31.0%)	1 (2.38%)	10 (23.8%)	2 (4.7%)	0.245
Rarely	0 (0%)	0 (0%)	0 (0%)	0 (0%)	
Sometimes	18 (42.9%)	3 (7.14%)	7 (16.6%)	8 (19%)	
Often	9 (21.4%)	2 (4.76%)	6 (14.2%)	1 (2.3%)	
Very often/Daily	2 (4.8%)	0 (0%)	2 (4.7%)	0 (0%)	
**Do they use additional oral hygiene aids?**					
Never	29 (69.0%)	3 (7.14%)	18 (42.8%)	8 (19%)	0.697
Rarely	7 (16.7%)	1 (2.38%)	4 (9.5%)	2 (4.7%)	
Sometimes	6 (14.3%)	2 (4.76%)	3 (7.1%)	1 (2.3%)	
Often	0 (0%)	0 (0%)	0 (0%)	0 (0%)	
Very often/Daily	0 (0%)	0 (0%)	0 (0%)	0 (0%)	
**How often do they brush their teeth with a toothbrush and toothpaste?**					
Do not brush teeth	4 (4.8%)	0 (0%)	2 (4.7%)	2 (4.76%)	**0.003**
Once a week	6 (14.3%)	0 (0%)	4 (9.5%)	2 (4.76%)	
Several times a week	11 (26.2%)	0 (0%)	7 (16.6%)	4 (9.5%)	
Once a day	11 (26.2%)	4 (9.52%)	5 (11.9%)	2 (4.76%)	
Multiple times a day	10 (23.8%)	2 (4.76%)	7 (16.6%)	1 (2.38%)	
**How does the patient maintain oral hygiene?**					
Independently	10 (23.8%)	1 (2.38%)	4 (9.5%)	5 (11.9%)	0.163
With assistance	28 (66.7%)	5 (11.90%)	19 (45.2%)	4 (9.5%)	
Does not maintain	4 (9.52%)	0 (0%)	2 (4.7%)	2 (4.7%)	

The values are presented as whole numbers and percentages. Chi-squared test or Fisher’s exact test. Significant values are in bold.

**Table 5:** Results of P-CPQ and FIS-8 questionnaires by subscales.

		Number of Carious Lesions			
		<5 (*n* = 6)	5–10 (*n* = 25)	>10 (*n* = 11)	
		M (Min–Max)	M (Min–Max)	M (Min–Max)	*p*
P-CPQ subscales	Oral symptoms	3 (2–12)	6 (0–10)	6 (4–9)	0.278
	Functional Limitations	6 (1–7)	6.5 (2–16)	8 (3–15)	0.380
	Emotional Well-Being	2 (1–9) ^a,b^	8 (2–12) ^a^	10 (3–13) ^b^	**0.004**
	Social Well-Being	2 (0–3)	0.5 (0–6) ^c^	3 (2–8) ^c^	**0.033**
FIS-8 subscale score	Parental Emotions	2 (0–4) ^d^	6.5 (0–16) ^d^	4 (3–11)	**0.020**
	Parental/Family Activity	0 (0–1)	3 (0–7)	1 (0–6)	0.103
	Family Conflict	0 (0.4)	2 (0–3)	2 (0–5)	0.971

Min—minimum value, Max—maximum value, M—median. The same superscript letters indicate significant difference between groups. ^a^ 0.028, ^b^ 0.001, ^c^ 0.015, ^d^ 0.017.

## Data Availability

Data supporting the findings of this study are available upon request.
